# Thermoregulation mechanisms and perspectives for validating thermal windows in pigs with hypothermia and hyperthermia: An overview

**DOI:** 10.3389/fvets.2022.1023294

**Published:** 2022-12-01

**Authors:** Jocelyn Gómez-Prado, Alfredo M. F. Pereira, Dehua Wang, Dina Villanueva-García, Adriana Domínguez-Oliva, Patricia Mora-Medina, Ismael Hernández-Avalos, Julio Martínez-Burnes, Alejandro Casas-Alvarado, Adriana Olmos-Hernández, Ramiro Ramírez-Necoechea, Antonio Verduzco-Mendoza, Astrid Hernández, Fabiola Torres, Daniel Mota-Rojas

**Affiliations:** ^1^Neurophysiology, Behavior and Animal Welfare Assessment, DPAA, Xochimilco Campus, Universidad Autónoma Metropolitana, Mexico City, Mexico; ^2^Mediterranean Institute for Agriculture, Environment and Development (MED), Institute for Advanced Studies and Research, Universidade de Évora, Polo da Mitra, Évora, Portugal; ^3^School of Life Sciences, Shandong University, Qingdao, China; ^4^Division of Neonatology, Hospital Infantil de México Federico Gómez, Mexico City, Mexico; ^5^Facultad de Estudios Superiores Cuautitlán, Universidad Nacional Autónoma de México, Mexico City, Mexico; ^6^Animal Health Group, Facultad de Medicina Veterinaria y Zootecnia, Universidad Autónoma de Tamaulipas, Ciudad Victoria, Mexico; ^7^Division of Biotechnology—Bioterio and Experimental Surgery, Instituto Nacional de Rehabilitación-Luis Guillermo Ibarra Ibarra, Mexico City, Mexico

**Keywords:** hypothermia, heat stress, infrared thermography, piglet, hog

## Abstract

Specific anatomical characteristics make the porcine species especially sensitive to extreme temperature changes, predisposing them to pathologies and even death due to thermal stress. Interest in improving animal welfare and porcine productivity has led to the development of various lines of research that seek to understand the effect of certain environmental conditions on productivity and the impact of implementing strategies designed to mitigate adverse effects. The non-invasive infrared thermography technique is one of the tools most widely used to carry out these studies, based on detecting changes in microcirculation. However, evaluations using this tool require reliable thermal windows; this can be challenging because several factors can affect the sensitivity and specificity of the regions selected. This review discusses the thermal windows used with domestic pigs and the association of thermal changes in these regions with the thermoregulatory capacity of piglets and hogs.

## Introduction

The regulation of body temperature in homeotherms is ensured by mechanisms of thermolysis and thermogenesis (1). Thermoregulatory adjustments can be induced by changes in environmental temperature and various physiological situations, including age, fasting, food intake, and stress conditions (2–4). Therefore, the evaluation of body temperature represents a valuable tool to monitor animals' physiologic status, welfare, and stress responses. Under stressful conditions, the activation of the sympathetic system and the hypothalamic-pituitary-adrenal axis (HPA) releases effector hormones such as catecholamine and glucocorticoids, respectively. Stress-induced hyperthermia, a condition triggered by the sympathetic system, consists of increasing core body temperature and the consequences on the thermoregulatory mechanism of animals (5).

The thermoregulation mechanisms of domesticated swine face challenges during all stages of growth due to certain anatomical-physiological characteristics of this species (6) that make these animals sensitive and susceptible to neonatal hypothermia, or hyperthermia in adulthood (7, 8), events that can trigger not only physiological alterations but also a predisposition to pathologies and mortality due to thermal stress (9). This impact productive and reproductive parameters and the quality of meat, milk, and other products of animal origin (10). The anatomical features that influence thermoregulation in swine include fine hair that helps reduce heat loss to a small degree under exposure to cold climates (11) and protects the skin from direct solar radiation in hot environments (12). The lack of functional sweat glands (13, 14) and low amounts of brown adipose tissue (BAT) at birth also impact the thermal response of domesticated pigs to diverse stimuli (15). However, some of these characteristics –like the lack of hair and heat-insulating fat behind the ears and near the sternum– make certain body regions candidates as thermal windows that can reflect heat exchange between the animal and its environment (16).

Infrared thermography (IRT) is used to measure the amount of heat that a body radiates (17, 18) as a result of vasomotor control that dilates or constricts peripheral capillaries (8). The discovery of this relationship suggested that IRT could be useful in veterinary medicine to evaluate circulatory changes caused by inflammatory or infectious processes, wounds, thermal stress, and stressful events (19). IRT could help perform detailed analyses of thermoregulation and the compensation mechanisms involved in returning to a state of homeothermy. However, achieving this goal requires identifying adequate anatomical regions and the information they can provide depending on their vasculature and location.

To date, the ocular, auricular, and nasal regions are most often employed to quantify the heat that animals dissipate or conserve (17, 20, 21). In species like swine and canines, appendicular regions are recognized as being more sensitive to temperature decreases due to prominent blood vessels that contract to prevent active heat loss (7, 22). IRT can detect changes of this kind in the surface vasculature of the skin, depending on the anatomical components of the region (15, 23). The use of IRT is, however, still controversial because studies have shown variability in indices of sensitivity and specificity, two parameters that can be affected by diverse factors, both internal (e.g., presence of hair, bare skin, and hair length, among others) (24), and external, that limit its use in veterinary medicine (11, 24).

This review aims to analyze (i) the thermoregulation mechanisms of piglets and adult pigs (hogs); (ii) the thermoregulatory adjustments that pigs of different ages confront; (iii) evidence for the use of various thermal windows with domesticated swine; (iv) the factors that affect their validation; and (v) possible limitations on applying IRT with this species.

## Review methodology

**Figure 1:**
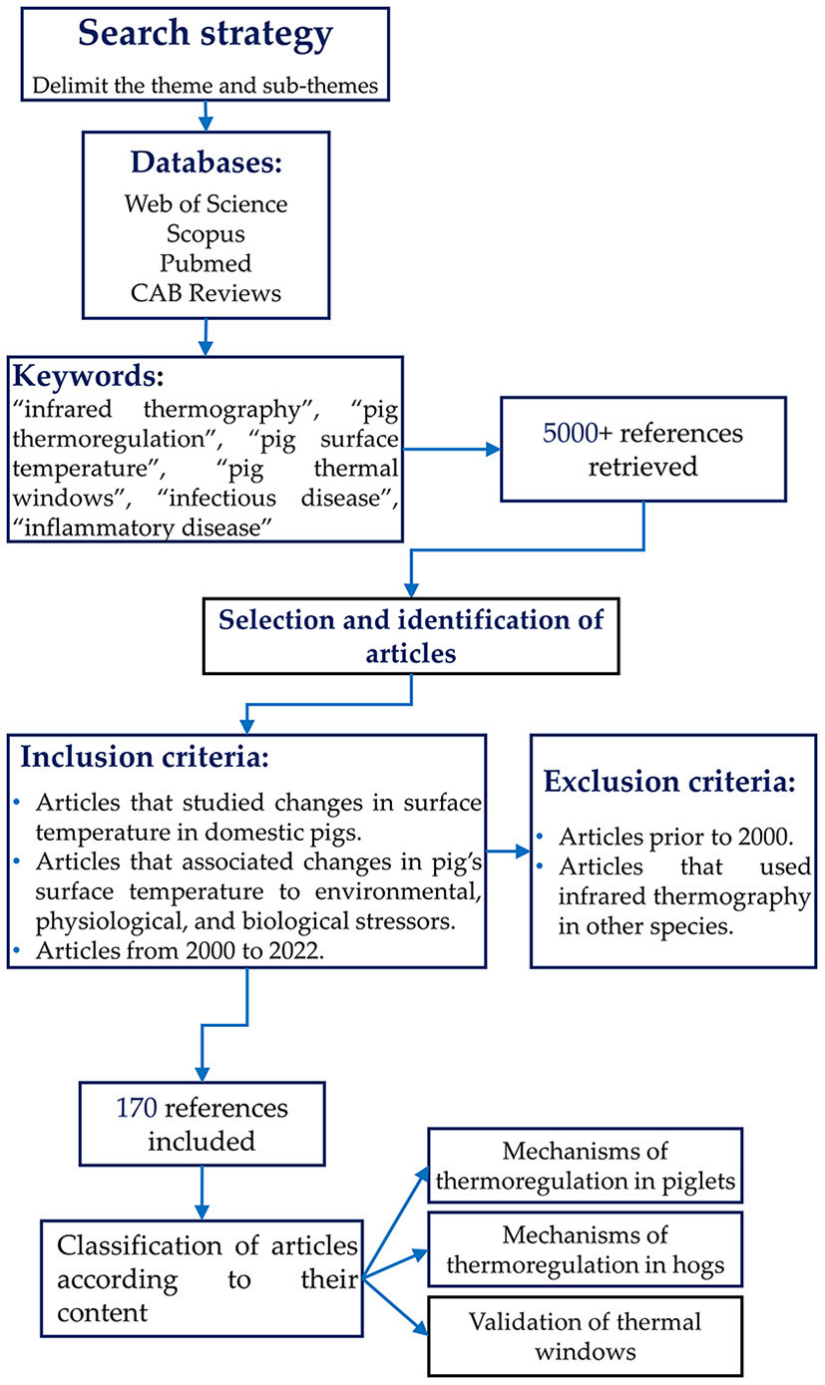
Flow chart showing the criteria used during the search of scientific articles for the overview.

The search was conducted in Web of Science, Scopus, PubMed, and CAB Reviews databases. The keywords used to find the literature were: “pig thermoregulation”, “pig surface temperature”, “infrared thermography”, “pig thermal windows”, and “infectious or inflammatory disease”. The inclusion criteria for the articles and books cited in the present review (*n* = 174) were those regarding the changes in surface temperature in domestic pigs in response to environmental, physiological, or biological stressors. In addition, articles between the years 2000 and 2022 were considered. Studies that did not meet the inclusion criteria or reported the use of infrared thermography in other species were excluded. Figure 1 describes the overall methodology for this review.

## Thermoregulatory contrasts between piglets and hogs, anatomical-physiological aspects, and differences in thermo-stabilization

The anatomical-physiological particularities that affect thermoregulation in pigs include scarce fine hair (25), apocrine glands distributed throughout the body, and the absence of eccrine sweat glands (26). However, these characteristics differ in distinct stages of development and growth. For example, due to the absence of BAT and microfibril mass and low fat and glycogen reserves, the piglet's thermoregulatory immaturity (27) helps explain why newborns are vulnerable to environmental challenges when the temperature is around 18–20°C. Piglets require the activation of compensatory mechanisms such as shivering thermogenesis, piloerection, and vasoconstriction in the first hours and days of life to impede heat loss from vital organs.

**Figure 2:**
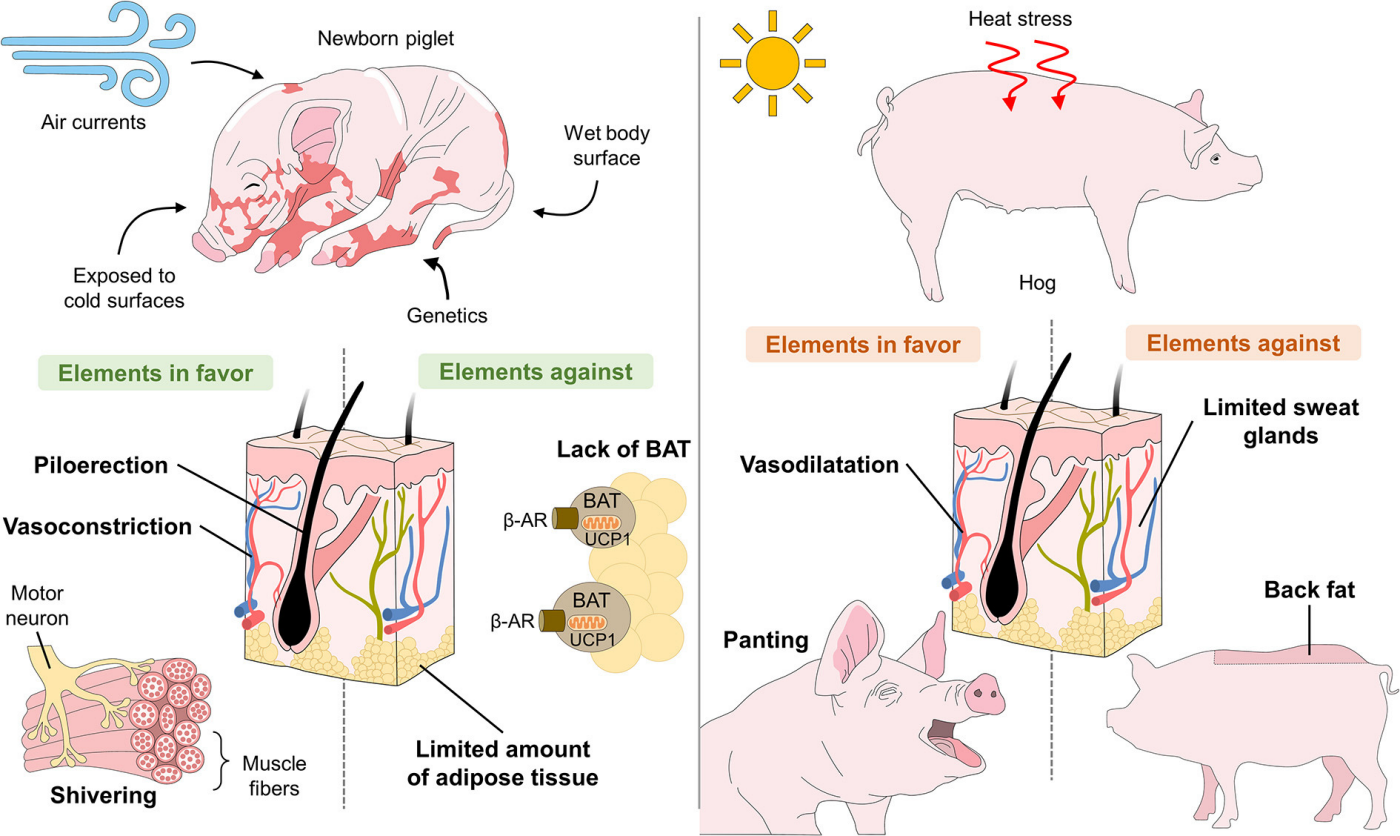
Anatomical-physiological challenges of piglets related to hypothermia and of hogs related to hyperthermia.

In hogs, in contrast, the disposition of adipose tissue is considered a factor that affects their heat-dissipating capacity, especially during exposure to high environmental temperatures. Hogs rely on peripheral vasodilation and panting to dissipate excess heat. However, due to their few functional sweat glands and the subcutaneous layer of backfat that impedes heat loss, they are susceptible to thermal shock that can also lead to death (Figure 2) (28–30).

Regarding behavioral thermoregulatory responses such as panting, pigs use a wide repertoire of changes to maintain their core temperature. For example, newborns huddle as the primary method to prevent heat loss, representing around 61.2–73.8% of their time (31). In sows, responses to cold environments include postural changes (sternal recumbency) (32), decreased locomotion (33), and an increase in food and water intake (34). In contrast, wallowing is the first reaction, when exposed to heat stress, and reduction in feed intake and motivation to search for warm places are frequently observed (27, 35).

A significant difference between piglets and hogs is the thickness of the skin and its supporting structures. The dermis and subcutaneous tissue of hogs measures around 3 mm, 21–26 μm (36). The thickness of the epidermis varies from one anatomical site to another, but ranges around 30–140 μm, which can be considered a barrier against cold temperatures (37). The skin is thicker and has numerous crests in the shoulders and snout but is thin around the dorsum and hips (38). In contrast, studies of one-day-old piglets report a skin thickness of 0.3–1.4 mm, a stratum corneum of 11–48 μm, and an epidermis of 33–68 μm (39). This influences their thermoregulatory capacity, tolerance to extreme climates, and interaction with the vascular anatomy that, in neonates, is similar to that of human skin (36).

Two other factors related to the skin are glandular structures and the presence of hair follicles. Piglets have a capillary: follicle ratio of 730/cm^2^, but reports on hogs estimate just 10.16 follicles/cm^2^ (36). The importance of these structures during the first days of life lies in their heat-conserving function generated by piloerection. In this regard, while swine are considered a species with scarce hair distribution, a study of Hampshire hogs weighing 100–120 kg reported the union of an arrector pili muscle with the outer root sheath (40) that supports the effects of piloerection and heat retention.

Another important characteristic that distinguishes swine from other species is the structure of their sweat glands. Unlike human sweat glands of the eccrine type and function mainly to cool the skin, pigs have apocrine glands that end in the epidermis, near hair follicles (41). They have been loosely described as simple, though the ones in the snout are considered more complex, as they are coiled and distributed in a ratio of 1:1 with respect to the follicles (40, 41).

Adipose tissue is another essential element for thermoregulation that differs significantly among swine of distinct ages. Neonate piglets have small fat deposits (around 15 g Kg^−1^) available in the abdomen and on the back (42), limiting their capacity to produce heat through food consumption (43, 44). Research has shown that piglets –which lack BAT at birth– use the process called “browning of white adipose tissue” (WAT) to perform non-shivering thermogenesis (45). Mersmann et al. (46) reported that the piglet's organism has 4% fat immediately postpartum. Other authors mention just 1%, but this can increase to 16.4% during the first 24 days of life (47, 48). In pigs, the fat-producing process begins in the fetal stage. From 0 to 68 days of gestation, the fat content has been reported to be around 0.06 g/d, but increases to 1.09 g/d at day 69 (47). As the age of the animal advances, the fat percentage increases to 9.3–24.3% (49), with an average of 30% of extra-muscular fat (44). This means that mortality by hypothermia is not a major challenge in hogs as it is in piglets. Instead, the increase in the thickness of the layer of adipose tissue reduces emissions of cutaneous heat into the environment through vasodilatation. This situation becomes complicated, however, when we consider the reduced functionality of sweat glands in swine, and the limited effectiveness of thermoregulatory behaviors like panting. These elements can cause imbalances when hogs are exposed to high environmental temperatures of 24–30°C and leave them susceptible to hyperthermia (known as an elevation of body temperature caused by an imbalance of thermoregulatory mechanisms that leaves them unable to eliminate heat at the same rate as it is produced) or heat stroke (50, 51).

Regarding the characteristics of the muscle required to perform shivering thermogenesis, skeletal muscle fibers are classified into “slow-twitch” (type I) and “fast-twitch” (type II with subsequent subtypes IIa, IIb, IIx), according to the isoform of myosin heavy chain and the contraction speed, where type IIb is the fastest (52). Vanden Hole et al. (53) determined that the percentage of type II muscle fibers in the pelvic limbs of piglets represents 95.58% of total muscle fiber. It has been reported that piglets have predominantly secondary muscle fibers, that these correlate positively with muscle weight (*r* = 0.39) (54), and that low temperatures of 15 ± 1°C in 21-day-old piglets increase the expression of type IIa fibers (55). In hogs, an immunohistochemical study of muscle tissue samples showed a higher proportion of type-II muscle fibers (56), which can increase quadratically with parity (3^rd^ birth, r2 = 0.44; 4^th^ birth, r2 = 0.54). Other studies of animals weighing 100 kg showed that the longissimus muscle expresses three isoforms of type II muscle fibers but not IIb, which were not observed in Lefaucheur et al.'s work (56). These fibers' distribution consists in islets of type I fibers surrounded by peripherally-located IIa- and IIb-type fibers (56). In hogs, the percentages of type I, IIa, and IIb fibers are 4, 8, and 88%, respectively (57), lower than the figures for piglets. The importance of the predominance of certain fibers is that while type II are fast, they require ATP production to initiate non-shivering thermogenesis, a substrate that may be limited in piglets. In contrast, type I rely on oxidative metabolism to thermoregulate (58), and a shift toward oxidative muscle can occur in piglets (55). Finally, reduced muscle irrigation, added to the absence of microfibril mass that controls the potency of muscular contraction, also compromises the mechanism of shivering thermogenesis (43), leaving piglets susceptible to neonatal hypothermia.

Concerning the metabolic mechanisms that pigs use for thermoregulation, glycogen reserves in skeletal muscle (around 30–35 g Kg^−1^) and liver are the main sources of energy in the first hours of life (43, 59). Glycogen concentrations in the skeletal muscle, front and hind legs, and liver of 32 neonates of a Topigs x German Pietrain breed were studied in the first 96 h of life. In that study, low birth weight in the piglets (body mass = 0.79 ± 0.26 kg) was associated with reduced glycogen utilization in the hind legs, as those reserves were not utilized until 8 h postpartum. Moreover, those newborns had as much as 50% less glycogen than normal-weight piglets (mass body = 1.37 ± 0.29 kg). An evaluation of the amount of glycogen in the liver showed that low-weight piglets did not utilize their reserves for 96 h, in contrast to the normal- weight ones that consumed half of that glycogen in the first 8 h of life (P = 0.0238) (60). Another energy reserve available at birth is glucose. Staarvik et al. (61) evaluated this in one-day-old piglets, finding average blood glucose concentrations of 5.48 mmol/l. Interestingly, they also found that male piglets had higher glucose levels than females, while those born in large litters had glucose concentrations as much as 0.07 mmol/l lower. Because glycogen and glucose are energy reserves that can be depleted quickly, consuming colostrum is essential for providing the energy newborns require for thermoregulation and the passive immunity needed to reduce the risks of low vitality (43, 59).

Recognizing the anatomical differences between newborn and adult pigs allows us to determine the resources each age group utilizes when exposed to environmental challenges like thermal stress, and also helps identify the responses that occur when those mechanisms are activated. Moreover, this information serves as a guide for planning strategies to prevent, diagnose, and manage affectations of the thermoregulation of swine in relation to their physiological stage of development.

## Thermoregulation in the newborn piglet

**Figure 3:**
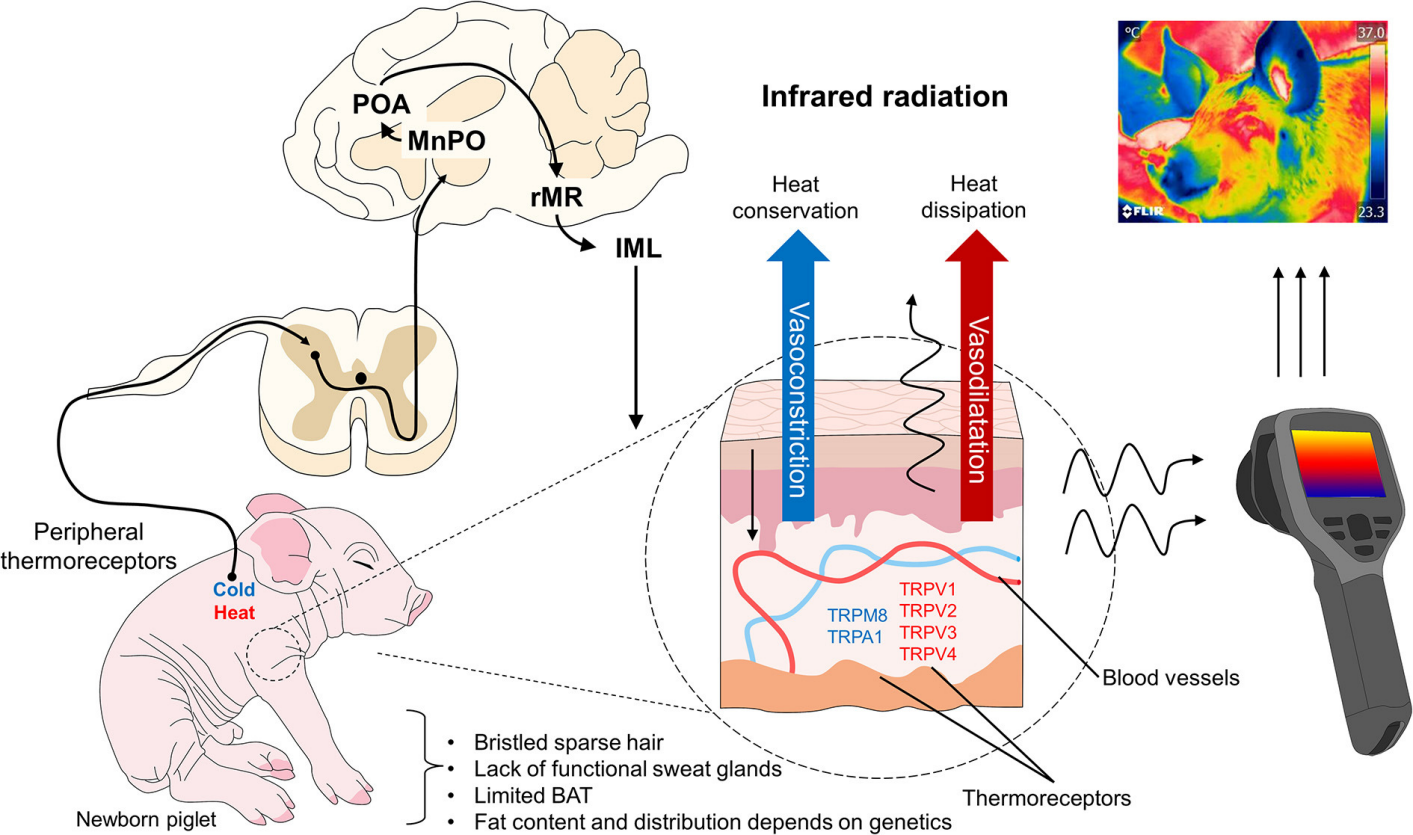
Thermoregulatory mechanism in domesticated swine. When peripheral thermoreceptors are activated to recognize cold or hot stimuli, the information is transmitted to the hypothalamus (MnPO) and other brain structures involved in the sympathetic control of peripheral blood vessels. When exposed to cold environments, these vessels vasoconstrict to conserve heat, but under exposure to hot temperatures, they vasodilate to dissipate heat. Both responses influence the amount of infrared radiation observable using techniques like IRT, though this also depends on the thermal window evaluated. IML, intermediolateral column; MnPO, median preoptic nucleus; POA, preoptic area; rMR, rostral medullary raphe; TRP, transient receptor potential vanilloid (V), melastatin (M), ankyrin (A).

At birth and during the first 48 h of life, piglets rely on shivering thermogenesis as their principal thermoregulation mechanism (62) (Figure 3) to compensate for low environmental temperatures of 20–22°C (43, 63), which can cause a drastic drop in body temperature, perhaps as great as 2°C (15, 64). The placental fluids (including amniotic liquid) that cover piglets together with the high body conductance and the high specific surface area at birth worsen this temperature drop due to the evaporation of 50% of those liquids that usually occurs in the first 5–30 min post-birth (65, 66). Hence, the main challenge for piglets is hypothermia. Their immature thermoregulatory center also plays an important role during birth; in fact, neuroimaging studies suggest that their thalamus and hypothalamus do not reach maturity until week 5 of life (67). This immaturity added to their relative physiological and metabolic immaturity, helps explain the incapacity of neonate pigs to thermoregulate when facing environmental challenges (15).

Because they do not have a mature thermoregulatory center (67, 68), newborn piglets first mobilize energy reserves (glycogen, fat, skeletal muscle, in descending order) to produce heat and survive in the extrauterine environment (7). The lack of microfibril mass that controls the potency of muscle fiber contraction (56) and the predominance of secondary muscle fibers help piglets produce heat through a muscular contraction so they can remain metabolically active. This may confer a particular advantage for achieving thermoregulatory success, but only in a limited way. Causes of thermoregulatory incapacity in piglets include reduced muscular irrigation, low mitochondrial mass responsible for the oxidative potential of muscle and, hence, the energy supply, and the level of bioavailability of carbohydrates as an energy source that leads to a biochemical modification of the oxidation of non-structural fatty acids, compromising the mechanism of shivering thermogenesis and leaving piglets susceptible to neonatal hypothermia (43).

The total amount of fat in piglets at birth and usually 42% of the limited muscle glycogen reserves are used in the first 12 h of life to satisfy its energy requirements. Nonetheless, the expenditure depends on factors such as body mass and vitality since piglets with low vitality have less muscle volume and liver glycogen stored at birth (69). Therefore, for this species, colostrum intake is critical for their survival. Reports show that colostrum provides around 5.4% of fat and 2.0% of lactose as bioavailable carbohydrates that support the newborn's survival. However, colostrum also gives piglets passive immunity by supplying immunoglobulin G, substantially reducing susceptibility to infections by agents like *E. coli* (70, 71). Under these conditions, piglets that are born weak with body weights < 800 g (72) are rarely able to ingest colostrum and milk, their two main sources of energy, because of (i) low energy reserves in their muscles that impede initial attempts to suckle, and (ii) insufficient insulation given their higher surface/body mass ratio that can lead to hypothermia (73).

- Inanition and hypothermia hinder locomotion, increasing the risk of crushing and death in the first 24 h post-birth. The piglet's organism may consume glycogen reserves in the liver or muscles during the first 6 h of life, striving to achieve thermoregulation despite hypoglycemia and limited glycogen reserves (74), but this can trigger metabolic acidosis, which that can end in coma or death by cardiac arrhythmia (7, 75). Studies show that piglets' 10–13% of perinatal mortality is due to underlying events like crushing and cold stress (43). Due to the reported correlation between piglet survival and the degree and duration of postnatal hypothermia (76), swine producers are interested in studies of hypothermia that may help prevent this condition that causes significant economic losses (77) as the leading cause of perinatal death (7, 78). The main threat to neonate piglets is a cold extrauterine environment that provokes active heat loss through evaporation and exposure to low temperatures and cold objects or fluids. Their limited thermoregulatory capacity exacerbates this due to scarce energy resources that are quickly depleted in such a challenging environment. Therefore, the timely recognition of hypothermia is crucial to avoid dire physiological consequences.

## Thermoregulation of hogs at the slaughterhouse

In contrast to piglets, hogs may be exposed to elevated environmental temperatures that affect growth, food conversion, reproduction, health, and welfare. Though hogs have a mature thermoregulatory center, they are susceptible to heat stress because they have few functional sweat glands (30/cm^2^) (79). Hogs radiate heat by convection and evaporation through vasodilatation by increasing their respiratory rate and changing postures (80). However, when temperatures exceed their body's thermoneutral zone (81), they cannot lose heat at the same rate as acquired. Stressful situations can also compromise the mechanism of peripheral vasodilatation and, hence, thermoregulation (82). Moreover, the effectiveness of the evaporative thermoregulatory response varies with the degree of humidity in the environment: the higher the humidity (50% or more), the less effective evaporative cooling will be. Hogs can suffer heat stress at lower temperatures than when the air is drier because less liquid evaporates from the respiratory tract and skin in humid environments (80).

It is important to understand that the magnitude of heat production and exchange in hogs depends, as well, on the stage of growth, gestation, lactation, diet, stocking density [1 *vs*. 2 m^2^/pig (83)], the air movement index, and convection and conduction methods (80). Animals in stages marked by high metabolic activity (e.g., early growth, lactation) tend to be more susceptible to heat stress, so sows, boars, and finishing hogs weighing over 50 kg may begin to experience the adverse effects of heat stress at temperatures that barely exceed 20°C (79).

Another critical characteristic of hogs is the layer of subcutaneous fat that insulates the skin but impedes thermolysis, leaving them especially sensitive to heat stress (84). The thickness of the hog's back fat is another relevant trait. Autochthonous, non-selected breeds like Iberian pigs have a thick layer of subcutaneous fat that can increase their sensitivity to high temperatures compared to leaner breeds (85). According to Usala et al. (86), the heritability of back fat depth is greater in conditions free of heat stress (*h*^2^ = 0.34 *vs*. 0.28). In line with these results, studies have proven that hogs weighing over 51.4 kg are more susceptible to heat stress than lighter animals, likely due to a limited heat-dissipating capacity because they have more adipose tissue (87). This corroborates the idea that certain anatomical features of hogs promote greater susceptibility to heat stress.

During transport to an abattoir, hogs may be exposed to stressors like dense loading densities and high ambient temperatures that can compromise homeothermy (88). When unable to maintain a balance between heat gain and loss, hogs suffer heat stress that can trigger myocardial and circulatory insufficiency, perhaps ending in death (89). The development of these insufficiencies is due to metabolic disorders like an increased flow of Ca^2+^ and consumption of adipose tissue, which foster a sustained temperature increase (90, 91) that can lead to oxygen deficiency, electrolyte imbalance, and oxidative stress caused by the activation of myeloperoxidase and eosinophil peroxidase enzymes which serve as indicators of ischemic changes (92).

### Adverse effects of hyperthermia for the health of hogs

In hyperthermic conditions, blood irrigation of the splanchnic tissues is channeled toward the periphery to dissipate heat. When this increase in body temperature is sustained for a certain time, it is classified as acute hyperthermia (93). Greater susceptibility to acute hyperthermia has been registered in hogs raised in tropical zones and during summertime in regions with temperate climates. This predisposition is greater than in other species; one of the factors is the lack of functional sweat glands. Heat dissipation occurs through two evaporative mechanisms (respiratory and dermal), but hogs dissipate < 50% of the heat produced *via* respiratory evaporation. Evaporation through the skin involves two types of processes: one passive, when water diffuses through the skin, and the other active, called sweating. However, hogs have a very low density of sweat glands (30/cm^2^) compared to species like bovines (800–2,000/cm^2^), and the few they have are not stimulated by heat stress, so little heat is lost by sweating. The second factor is subcutaneous fat. Heavier hogs (e.g., gestating or lactating sows and hogs of commercial weight) require greater energy consumption in relation to their productive stage but also present a lower heat-dissipating capacity because of a low surface: mass ratio (volume) and larger amounts of fatty subcutaneous tissues that impede heat dissipation (93, 94).

At the organ level, during hyperthermia, the gastrointestinal system is affected by hypoxia of the intestinal mucosa because blood flow (nutrients and blood) is diverted to the periphery. This damages the intestinal tract by reducing the height of the villi and the depth of crypts, increasing permeability, and intensifying the inflammatory response (93). There are also reports of tachycardia, hypertension, and supraventricular or ventricular arrhythmias, possibly indicative of cardiovascular damage after an hyperthermia episode (94, 95). Chen et al. (96) identified polymorphonuclear leucocytes in heart tissue sections from hogs affected by heat stress, suggesting that the animals were in an acute stage and may have developed myocardial lesions. As environmental temperatures rise, efficiency is compromised because maintaining a stable body temperature becomes the priority, so nutrients are channeled to achieve euthermia, pushing the synthesis of products (meat, milk) to a second plane. Hyperthermia also affects numerous intracellular signaling pathways responsible for survival and productivity (97). The effects of high temperatures on production can vary widely. Sows may present late-onset puberty and long intervals between weaning and estrus. In addition, the proportion of impregnated sows that give birth may be low. In boars, seminal quality may decrease (94), causing economic losses in the industry due to reduced efficiency, higher outlays for veterinary care, low meat quality because of increased lipids and reduced proteins, and higher mortality, especially especially under challenging stages for dissipating heat (e.g., gestation, fattening) (97).

Unfortunately, common hyperthermia is not the only condition threatening existing swine populations, for recent increases in demand for pork have intensified the breeding of genetic lines that reach the meat-production stage more quickly. While this process produces pigs with a higher muscle-to-fat ratio, it also brings undesirable traits, including a recessive, monogenic hereditary autosomal syndrome called malignant hyperthermia or porcine stress syndrome (PSS) (98). This pathology is described as a hypermetabolic reaction of skeletal muscle caused primarily by alterations of calcium channels in skeletal muscle cells and the central nervous system (Purkinje cells). If untreated, it can be fatal. Studies have described a mutant ryanodine 1 receptor gene (RYR1) that can cause episodes of malignant hyperthermia under adverse or stressful conditions, such as high environmental temperatures, intense exercise, reproductive activity, transport to the abattoir, or the application of inhaled halogenated anesthetic agents (95). Unlike the hyperthermia described earlier, which develops progressively, malignant hyperthermia is characterized by a sudden increase in body temperature and metabolic index. It usually ends in death. The dysfunction of calcium channels in skeletal muscle cells caused by mutations of the RYR1 gene produces a high concentration of intraplasmatic calcium because the Ca^2+^- releasing channels anchored to the membrane of the endoplasmic reticulum stay open (99). This disorder has become more rare due to the extensive removal of carriers of this gene mutation (100–102); the environment and the facilities where the animals are kept continue to be a factor in the development of disrupted thermal states.

The evolution of PSS passes through 3 phases. The first is marked by increased skeletal muscle metabolism, greater oxygen and glucose consumption, hypercapnia, and higher CO^2^ concentrations at the end of expirations that lead to hyperventilation. As energy supplies are consumed, the hog presents hypoxemia and hypoglycemia. Then, attempting to revert this, it intensifies glycolysis, which can produce lactacidemia. In the second phase, local hypoxia, an acid-base imbalance, and fever lead muscle cells to suffer necrosis. Combined with edema, this can trigger compartment syndrome. The rupturing of skeletal muscle fibers releases their content into the bloodstream (rhabdomyolysis). The electrolytes that enter the blood can cause fatal organ damage, while increases in plasma myoglobin and creatine phosphate kinase (CK) levels can trigger acute renal insufficiency. In stage three, the hog suffers systemic metabolic disorders, core hyperthermia, arrhythmia, heart damage, and continuous rupturing of myocytes that, finally, cause disseminated intravascular coagulation (DIC), multiple organ failure and death (95).

**Figure 4:**
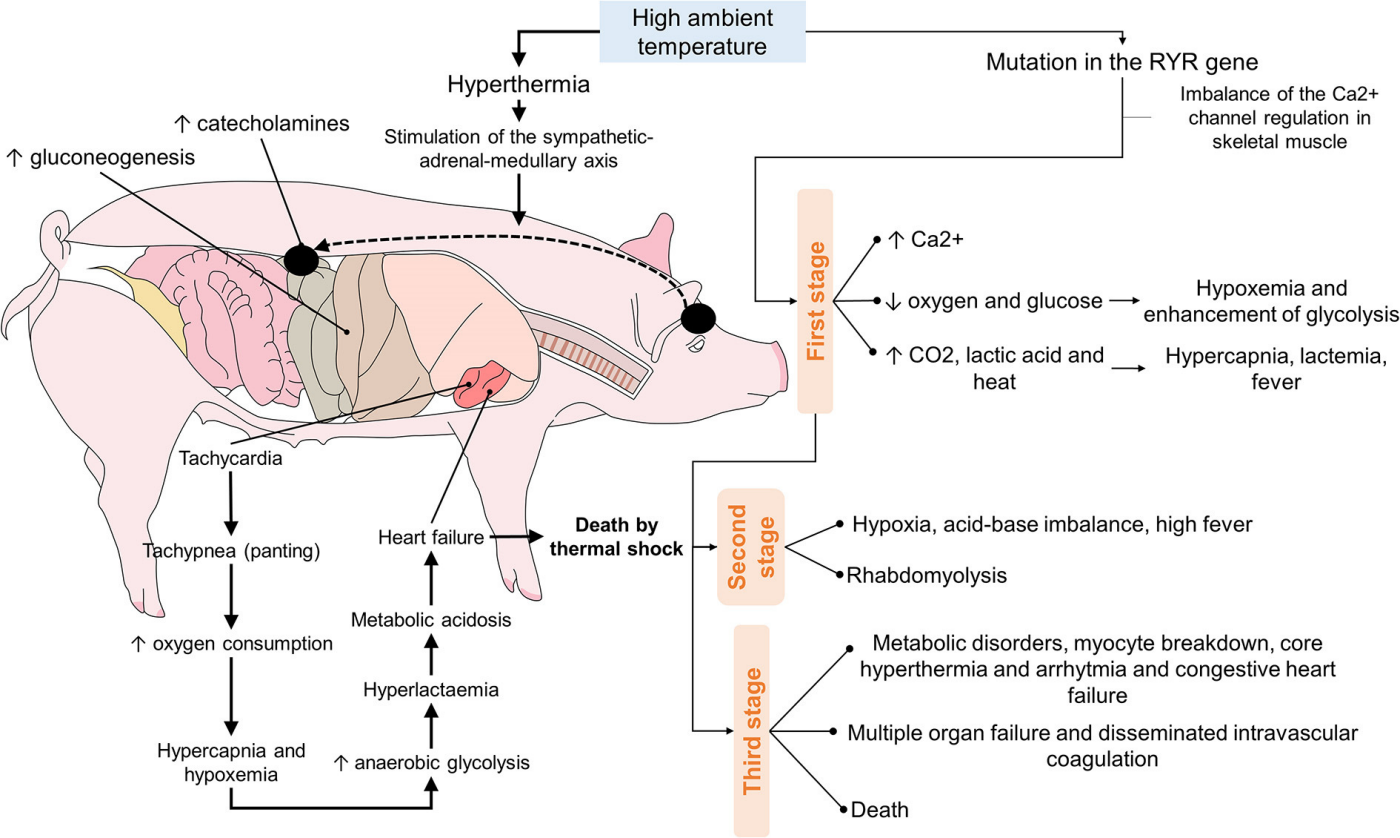
Sequence of the events that culminate in the death of hogs due to the effects of high ambient temperatures.

Nevertheless, even hogs that do not present the RYR1 gene mutation can die of heat stroke. A hog exposed to excessive heat may develop hyperthermia and activation of the sympathetic-adrenal-medullary axis (SAM), as in other stressful situations. Activation of this axis culminates in increased catecholamines synthesis and secretion, increasing gluconeogenesis, tachycardia, and tachypnea (103). It is important to understand that tachypnea –that is, panting– helps eliminate heat by evaporating liquid from the respiratory tract (27). Hyperventilation and greater oxygen consumption lead to hypercapnia and hypoxia, aggravated by hyperlactatemia due to increased anaerobic glycolysis. Finally, all these conditions together produce metabolic acidosis that affects heart function and, possibly, death (Figure 4) (103).

In recent years, besides changes in pig farm management and handling, alternative tools have been proposed to aid in assessing the thermal states of piglets and hogs. One of these complementary tools is IRT (104, 105).

## Infrared thermography (IRT) as a tool to evaluate the thermal and physiological state of swine

IRT measures surface temperature non-invasively by quantifying the radiation emitted by a biological body that reflects modifications in peripheral blood circulation (18, 23). Using IRT with humans and animals requires identifying body regions with specific characteristics called thermal windows, characterized by a high density of blood capillaries, arteriovenous anastomosis, and glabrous skin. In veterinary medicine, the eye, auricular pavilion, and tail are usually the foci of IRT used in species like rats, dogs, and large ruminants (23, 75, 106). However, the usefulness of IRT has been questioned because physiological and circulatory responses can differ among individuals and species depending on the region chosen due, for example, to the properties of hair in dogs or the skin thickness of large ruminants (11).

In newborn piglets, the entire body is considered a thermal window because of its low-fat content. In contrast, in adults, as the fat levels increase and reduce heat emissivity (50, 51), the main thermal windows proposed are the eyes (50, 107), ears, vulva, udder (108), armpit, back, shoulder, and snout, all of which have successfully been correlated positively with average rectal temperature values. However, the sites with reports of greatest reliability and accessibility are the base of the ear, shoulder, and udder (50, 108). Scientific evidence for thermoregulation in swine demonstrates that environmental temperatures play a particular role, so any attempt to determine the efficiency of thermal windows must consider this external factor.

To date, IRT has been used to detect febrile viral states, diagnose pathologies of public interest in pigs (109, 110), or inflammatory processes such as lameness in reproductive females (111).

### Thermal window: Orbital zone (periocular area and lacrimal caruncle)

**Figure 5:**
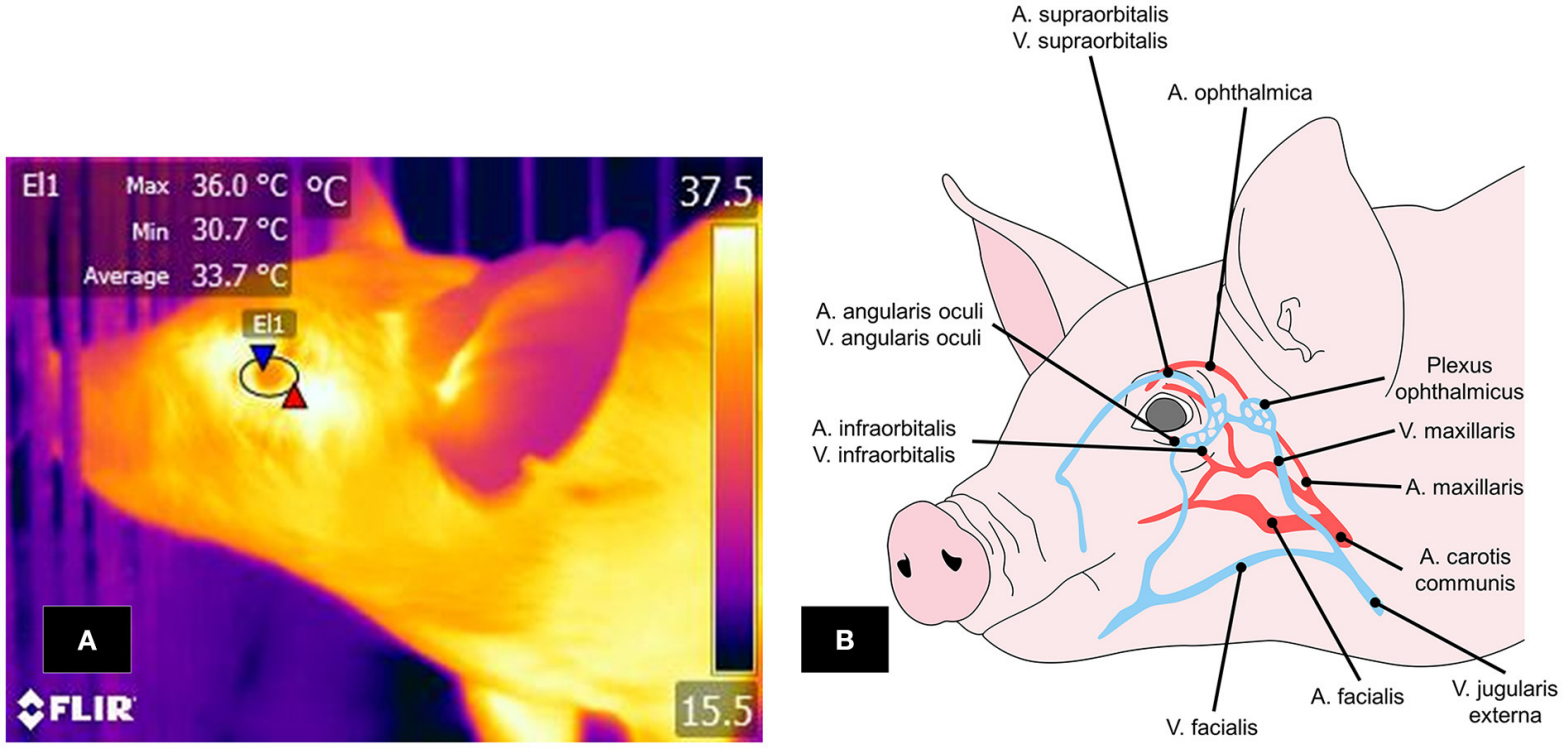
Ocular window in pigs. **(A)** The periocular window is outlined by an ellipse (El1) that surrounds the entire ocular surface, including a few millimeters of the upper and lower eyelids. **(B)** Irrigation to the ocular region, mainly by the supraorbital and infraorbital arteries and veins, which are branches of the maxillary artery and vein. IRT can detect changes in the muscle tone of these vascular structures and the heat radiated through them.

Two thermal windows have been identified in the orbital region: the lacrimal caruncle and periocular area. The infraorbital and supraorbital arteries pass through this region as ramifications of the maxillary artery, supplying blood to the eyelids and ocular muscles (112). The infraorbital artery also has ramifications of the facial nerve, sympathetic fibers that permit vasomotor control by activating the autonomous nervous system (ANS) (113) (Figure 5).

The ocular window has been studied mainly to determine the thermal state of animals and evaluate body temperature non-invasively due to its contiguity to the CNS (114, 115). This was proven in a study by Barbieri et al. (107), who evaluated if this thermal window could help to determine body temperature in 108 pigs. They obtained temperature readings with IRT and a calibrated digital rectal thermometer. The mean rectal temperature was 38.9 ± 0.4°C (min = 37.9°C; max = 40.1°C), while the mean ocular temperature was 36.7 ± 0.1°C (min = 34.8°C; max = 38.8°C). In general, the authors observed a moderate correlation between ocular and rectal temperatures (*r* = 0.58, *P* < 0.01), meaning that surface ocular readings and rectal values increased; however, the correlation varied from strong in recently-weaned pigs (*r* = 0.73, *P* < 0.01) to weak in fattening animals (*r* = 0.23, *P* < 0.05). This correlation may be because food intake and the age of the individual are factors that affect the animal's thermoregulation level. This situation has become evident in piglets, where the colostrum intake improves their thermoregulatory capacity and compensates for the limited availability of energy resources at birth. In older animals, food intake helps to maintain the temperature within the thermoneutrality zone which tends to be narrow. Therefore, during a state of hyperthermia, Therefore, the pig decreases feed intake (7, 43). These studies demonstrate the validity of IRT for determining thermal states in animals when significant temperature alterations occur. Results coincided with those mentioned in a scientific review, which stated that temperatures recorded in the ocular region reflect the circulatory control present in that area and its close relation to the ANS (8).

The physiological explanation of the temperature increase is based on greater cardiac activity in response to the catecholamine neurosecretion (adrenaline, noradrenaline) stimulating sympathetic nervous system activity (SNSi), affecting body temperature, a condition called stress-induced hyperthermia (88). Since this phenomenon induces a state of hyperthermia, IRT could serve to detect the increase. Pulido-Rodríguez et al. (116) evaluated the relation between temperatures in the ocular region (thermographic images taken while keeping a distance of 0.50–1 m) and cortisol levels in 66 piglets for 7 weeks post-weaning. They found that in the first 2 weeks, saliva cortisol levels and ocular temperatures presented a strong positive correlation (*r* = 0.89, *P* < 0.05) and concluded that higher temperatures in this region serve as a reliable indicator for detecting stress-induced hyperthermia and adrenal response.

These findings were confirmed by Machado et al. (117) in their study of 192 hogs transported for 170 km, where they recorded rectal and ocular temperatures (with a fixed distance of 0.50 m between the animal and the observer), saliva cortisol levels, respiratory rate, and lactate levels, while also comparing the influence of the upper and lower transport levels inside the vehicle. They observed that the average post-transport temperature in the animals at the upper level was higher than those below (upper = 39.9°C, lower = 38.1°C; P = 0.022). That finding coincided with higher lactate levels (upper = 61.63 mg/dL, lower = 58.26 mg/ dL; *P* < 0.001) to indicate that temperature readings provide information on both the thermal state of animals and their welfare levels (51, 114).

When considering hyperthermia, Weschenfelder et al. (118) evaluated 258 hogs prior to sacrifice to determine the effect of the pre-slaughter process and the influence of meat quality. Those authors measured ocular temperatures using IRT (keeping a distance of 0.25 m) and the pH of mass in the long dorsal, semi-membranous, and adductor muscles. Although observations showed that ocular temperatures correlated with plasma lactate levels (*r* = 0.20, *P* = 0001), with pH at 1-hour postmortem (*r* = 0.18, *P* = 0.03), and with pH of the semi-membranous muscle (*r* = 0.20, *P* = 0.02), the degree of correlation was weak. The authors concluded that the accuracy of IRT should consider elements such as dirt, hair, and humidity. Results coincided with those mentioned in a scientific review, which stated that temperatures recorded in the ocular region reflect the circulatory control present in that area and its close relation to the ANS (8).

The use of the ocular region to detect hyperthermia could also be used to identify other conditions, such as febrile states. Mota-Rojas et al.'s scientific review (104) observed that because IRT is used to detect anatomical regions with increased radiated heat, the ocular area could be utilized to recognize febrile states caused by infection. During infection, the physiological difference that causes the temperature increase during the fever must be the release of cytokines like interleukin-1, interleukin-6, and prostaglandin. Those substances are classified as pyrogenic molecules that stimulate the preoptic area of the hypothalamus to increase body heat (104).

The effects of febrile states were proven in a study of 124 Landrace x Yorkshire pigs diagnosed with *Actinobacillus sp*. infection. The researchers found that IRT taken at a distance of 0.50–1 m predicted body temperature increases of 0.80 and 0.35°C (119). That evidence was confirmed byLoughmiller et al. (120), who evaluated febrile responses in 28 pigs using IRT (keeping a distance of 2 m). In the six animals inoculated with *Actinobacillus pleuropneumoniae*, the authors observed a significant interaction between IRT temperature readings and inoculation with the pathogenic agent (*P* < 0.001), as temperatures were significantly higher than in the control animals (infected = 39.6 ± 0.3°C *vs*. non-infected = 32.6 ± 0.3°C, *P* < 0.05). These results demonstrate IRT's sensitivity for detecting febrile states and the need to analyze this thermal window for evaluating body temperature.

Febrile responses are not necessarily associated with pathological conditions in animals, as they can also occur as biomarkers of immune responses to vaccination (109). This was proven in a study of vaccinated pigs in which IRT (thermographic images of a group of pigs were taken keeping a distance of 2 m) detected a temperature increase of 1°C in the 3–8 h post-injection (121). Scientific evidence thus conclusively shows that the ocular region has useful clinical applications for treating sick animals. Other authors, however, argue in favor of using the lacrimal caruncle region because it is innervated by sympathetic fibers and responds to ANS activity and, therefore, could be a biomarker of stress (23, 24).

The thermal window of the lacrimal caruncle has been utilized in studies like one by Lonardi et al. (122) that evaluated 2500 Large White and Belgian Landrace pigs' eye temperature (keeping a distance of 0.8 m between camera and skin surface) under two conditions: castration and non-surgical handling. Those researchers observed that the temperature of the lacrimal caruncle was higher in the first group than in the one that received only handling at 3 h post-surgery (35.6 ± 0.08°C *vs*. 35.4 ± 0.09°C, *P* < 0.05). They also found that the temperature increase had a weak correlation with rectal temperatures (*r* = 0.31, *P* < 0.01), perhaps indicating that the ocular window is more specific for painful conditions or stress in swine. However, it has also been suggested that this window may be more helpful in identifying temperature decreases caused by increased sympathetic activity. This precise increase that triggers neurosecretion of catecholamines which causes vasoconstriction of the capillaries to reduce heat radiation, as has been reported in other species (18, 123). The behavior of this window continues to be studied, but its clinical utility is undeniable, as is the assistance it offers veterinarians. For this reason, it is necessary to gather additional data to efficiently demonstrate the information obtained from the ocular region in order to achieve a strong validation.

Another important factor is the thickness of the skin because it influences heat radiation. Moreover, the capacity to dissipate heat differs among anatomical sites like the ear, eye, back, and buttocks (108). This situation explains that thicker skin impedes dissipation, in contrast to thinner-skinned animals like the water buffalo (124–126) (pig: 30–140 μm vs. water buffalo: 50–115 μm) (24, 38). Zhang et al. (127) suggest that these variations are related to the reflection of radiant energy and emissivity, which vary at different anatomical sites, given that infrared cameras detect infrared radiation of 0.75–1.4 μm and thermal radiation of 8–15 μm (128). For example, the recommended emissivity values for pigs range from 0.94 to 0.98 (129, 130). However, the thoracic limbs and eyes values are 0.94 and 0.93, respectively. Therefore, the anatomical characteristics of the pig must be considered to achieve precise evaluations of surface temperatures in real-time. It is also essential to emphasize that other techniques must confirm IRT diagnoses. Thus, the best approach is to use IRT and other tools to optimize pig farm management.

### Thermal window: Auricular

**Figure 6:**
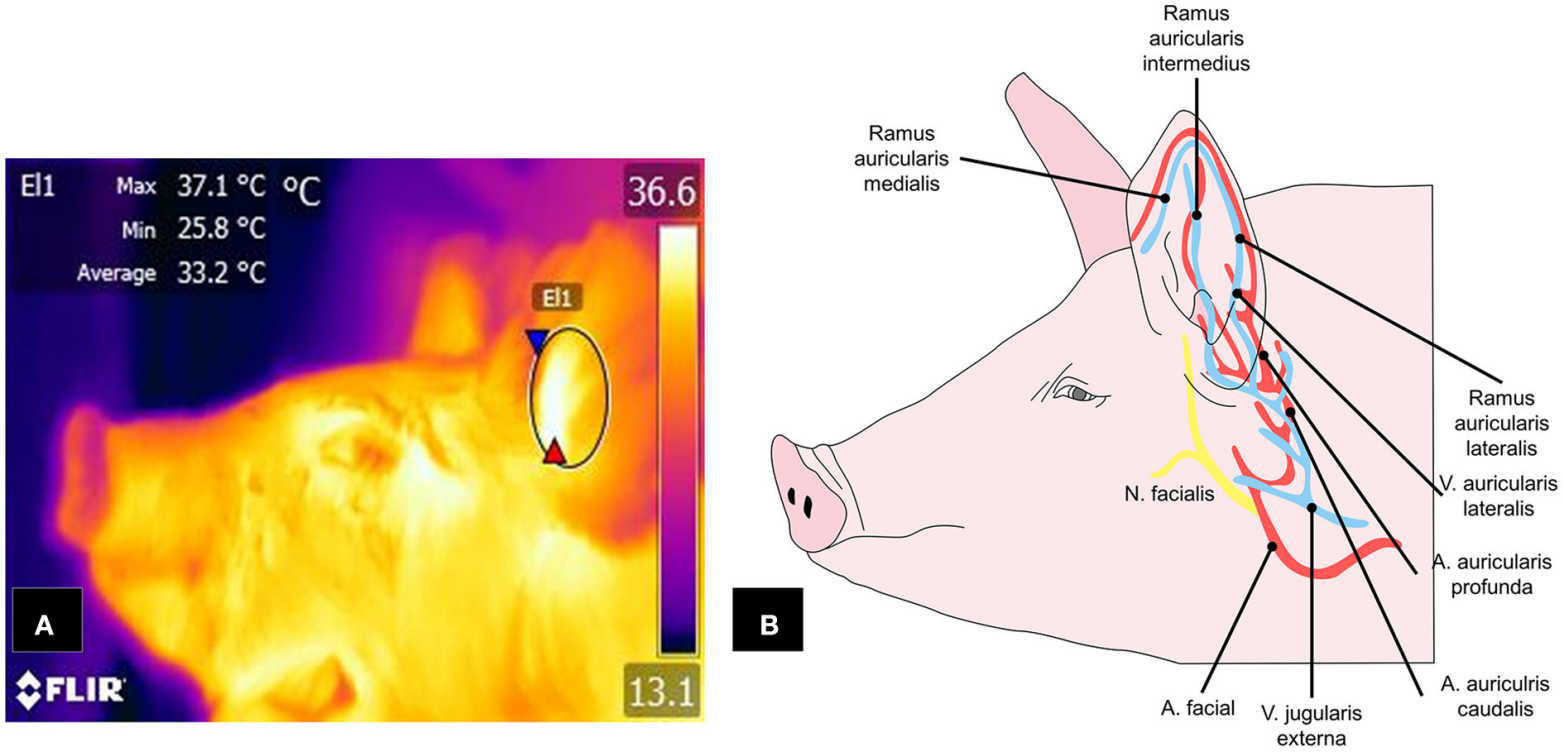
Auricular window in pigs. **(A)** The window is delimited by an ellipse (E1) that includes the central cartilage. **(B)** Circulation from the facial artery and external jugular vein, with their respective branches, spreads into the *ramus auricularis medialis, intermedius*, and *lateralis*, which are responsible for transporting blood to the ear pavilion and that respond to activation of the ANS.

Another area that has been shown to satisfy the characteristics for consideration as a thermal window is the auricular region. Figure 6 shows the vasculature of the auricular pavilion, mediated by the caudal auricular artery (*auricularis caudalis*) divided into three regions: lateral (*lateralis*), intermediate (*intermedius*), and medial (*medialis*) (131). Innervation of this region depends on the auriculopalpebral nerve (*auriculopalpebralis*), a ramification of the facial nerve (*facialis*) (112).

Contrary to observations of the ocular window and its capacity to recognize hyperthermic states, the auricular area has been used to recognize hypothermia in piglets (7). In animals aged 1–13 days, the surface temperature of the auricular region presented a strong correlation (*r* = 0.89, *P* < 0.01) with rectal temperatures (132), so it may be indicative of the information provided by the pavilion. This theory was posited by Schmitt and O'Driscoll (133) to validate IRT for evaluating temperature in piglets. The authors assessed 67 animals by recording temperatures of the ear and back. Results showed that, in general, the temperature of the ear was lower in low-weight animals (1.5 kg = 35.2 ± 0.36°C *vs*. 1.74 kg = 36.5 ± 0.35°C, *P* < 0.001). This finding was similar to observations of low rectal temperatures in animals with severe growth delay (severe = 35.8 ± 0.46°C *vs*. mild = 37.2 ± 0.42°C, *P* < 0.05).

Some authors concur with this idea, according to Schmitt et al. (134), who evaluated the thermoregulatory capacity (taking thermographic images of the auricular window at a distance of 1 m) of two lines of piglets with differences in food consumption. They found that post-birth, the high-consumption animals had lower temperatures than those with low consumption (24.7 ± 0.37°C *vs*. 26.3 ± 0.36°C, *P* < 0.005). The explanation offered is that exposure to cold induces heat production by metabolic thermogenesis, which intensifies consumption of energy resources like BAT, while in neonates with scarce energy resources, food consumption increases to compensate forand regulate temperature (27). This explanation makes it possible to argue that the performance of thermoregulation in the first weeks of life, up to weaning –when the temperature is strongly influenced by factors like humidity and the animal's weight and average food consumption– corroborates the importance of monitoring this condition (7, 135). Vascularization and innervation of the auricular window responding to changes in ANS tone as an activation of the SNSi, as we saw in the section on the ocular window. This was posited in a study by Yañez et al. (136) based on an evaluation of 64 piglets weaned with or without social disruption who did or did not receive environmental enrichment. The study animals received environmental enrichment in suspended ropes, aromatized bottles, toys, and balls, while the control group did not. The authors found that temperatures in the auricular pavilion did not present changes under any of the treatments but that the temperature of the lacrimal caruncle was 1.7°C lower in the animals that did not receive enrichment but experienced disruption of the social order (*P* < 0.05; at a uniform distance of 1 m). In contrast, the animals without disruption and with environmental enrichment had higher lacrimal caruncle temperatures than controls (*P* < 0.05). From a comparative perspective, these results demonstrate that the meaning of the thermal response observed differed with the condition to which the animals were exposed and that areas like the ocular region, specifically the lacrimal caruncle, can help evaluate acute stress responses. In contrast, the ear region may indicate an animal's overall thermal state (137).

The results just reviewed contrast to those of Rocha et al. (138), who set out to validate anatomical sites like the ear, ocular region, neck, and rump (taking thermographic images at a distance of 1.50 and 2.6 m) in 120 pigs under two treatments, in this case, rough *vs*. gentle handling. They evaluated physiological parameters like heart and respiratory rate and body temperature, in addition to surface temperatures at the anatomical sites chosen for study. Observations included increased heart rate, body temperature, and saliva cortisol levels in the animals that received rough handling, with temperature increases in the ocular region and the ears of 7 ± 0.29°C and 5.86 ± 0.46°C, respectively. It is important to mention that those authors found moderate correlations for the regions of the eye and ear as saliva cortisol levels increased in the animals handled roughly (*r* = 0.49 and *r* = 0.50, respectively, *P* < 0.001). This study provides sufficient evidence to validate the lacrimal caruncle and ear region as areas where stress responses in pigs can be evaluated. Some authors, however, mention that readings from these areas can be affected by other factors, such as the distance at which they are taken, the specific region evaluated, humidity, and skin thickness, so these elements must be considered when choosing and evaluating anatomical sites (24, 132).

The auricular region, then, can be considered a thermal window due to its vascularization, which makes it especially sensitive to changes in environmental temperatures. It is a good site for detecting hypothermic states in neonates. Its innervation plays a vital role in changes in the diameter of the capillaries that can respond to catecholamine levels under stressful conditions, but it has the additional advantage of having been validated, like the ocular window (138).

### Thermal window: Nasal

**Figure 7:**
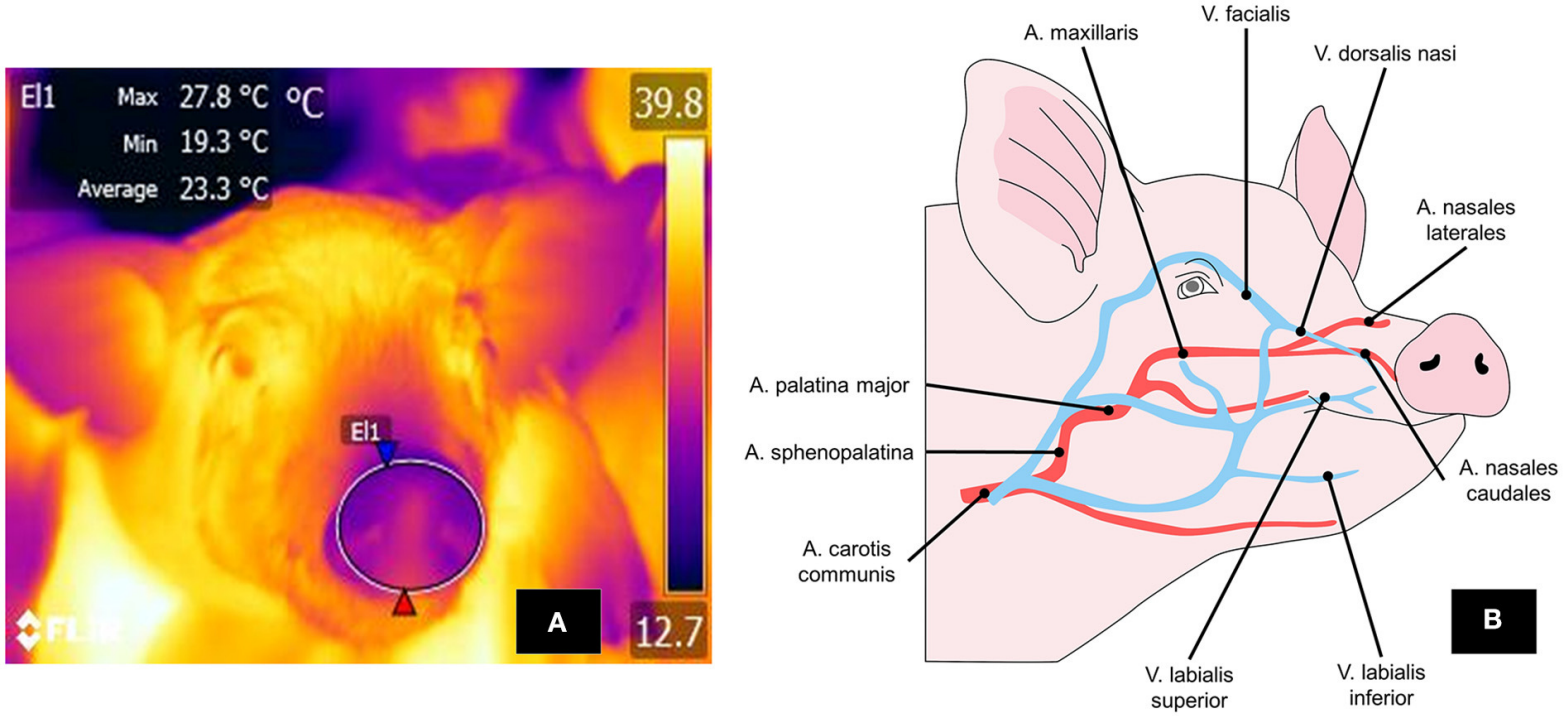
Thermal nasal window. **(A)** To mark the perinasal window, a large circle is drawn around the entire snout with the upper lip as its ventral limit. **(B)** The nasal region and vasomotor changes there depend on circulation through the lateral and caudal nasal arteries and the *dorsalis nasi* vein, a branch of the *facialis* vein. These structures respond with vasodilation or vasoconstriction, according to the stimulus perceived.

The nasal thermal window, located in the region of the same name, receives blood flow from the nasal artery, a ramification of the maxillary artery (*artery maxillaris*) (139) (Figure 7). This window has been used to evaluate the temperature of the nostrils because they can lose heat by evaporation through respiration (140).

Research on humans has analyzed the effects of stressful psychosocial and physical conditions on vasomotor control of the nose (141). The blood supply to the nose region is sensitive to activation of the SNSi, and, therefore, vasoconstriction of surface blood capillaries, translates into the reduced blood flow and less heat irradiating into the environment (136). However, interpretations of surface temperature readings from the nostrils must consider that respiration produces vapor. While the presence of this inspired vapor is undoubtedly a drawback regarding the usefulness of this thermal window, it can also be considered an advantage because it permits quantifying other parameters, such as respiratory rate. Ricci et al.'s (19) 7-day study of 26 pregnant sows collected data on the parameters of respiratory rate and rectal temperature together with IRT readings from the nasal window (from a distance of 1 and 1.5 m) in the morning and at night. The animals showed a moderate correlation between nasal temperature and respiratory rate (*r* = 0.350), meaning that as the temperature of the nasal region increases, the respiratory rate increases due to tachypnea as a thermoregulatory compensation mechanism, although there is no direct relationship between these two variables.

Similarly, Jorquera-Chavez et al. (142) conducted a pilot study with pigs infected with *Actinobacillus pleuropneumoniae*. They determined that the respiratory rate in the sick animals, evaluated by IRT of the nasal area, increased by an average of 10 breaths/min (rpm) compared to the healthy animals. This coincides with reports on bovines, where the use of this window has been explored as a non-invasive way to record respiratory function (24). However, this application requires additional study because free-moving pigs infected in a normal on-farm environment have not been tested (142). Furthermore, as was reported in a study of 24 male growing pigs exposed to heat stress (at 34°C), to date only rectal temperature has been associated efficiently to determine respiratory rate, with R2 values of 0.997 and 0.993 for acute and chronic heat stress, respectively (143). These findings reaffirm that said thermal window could be associated with the causal factors of heat stress.

Taking into account that rectal temperature is considered the gold standard for quantifying body temperature, Malmkvist et al. (144) analyzed the influence of the thermal environment (15°, 20°, 25°C) on sows during farrowing and lactation based on thermal responses in the head and body regions, as well as in rectal temperature. In their analysis of the data, the authors found that the temperature of the snout increased gradually in relation to the temperature of the holding room, as they recorded average values of 33°, 35°, and 36°C, respectively. They further reported that the rectal temperatures behaved similarly, showing influence by room temperature (38.0°, 38.7°, and 39.0°C, respectively). However, the nasal temperature of the sows maintained a weak correlation coefficient of 0.10 with rectal temperature, a value lower than those obtained for the ocular window (0.24) and udder (0.36). It is worth mentioning that the temperature increase of the snout was accompanied by tachypnoea, as rpm increased from 29 to 58 in rooms maintained at 15° and 25°C, respectively. Due to these findings, it is possible to affirm that the nasal window could be used to associate it with respiratory rate, not with rectal temperature, but it is subject to environmental variations and the effect of social stress factors (145).

These data provide a better understanding of the influence of the environment on the thermoregulation mechanisms of swine in different production stages. In the case of 16 guilts (primiparous sows), pregnant sows, lactating sows, and suckling piglets, IRT was evaluated in seven body regions, including the nose. In general, in all groups, this window showed the lowest temperature values, with an average of 33°C, in contrast to the temperatures registered in the ear root or the tail base (36.2° and 37.1°C, respectively). Their findings led the authors to suggest that IRT could serve to frequently monitor environmental temperatures or early disease states (146) since temperatures reflect the health status of animals (147).

### Other thermal windows of the body

Pigs are endothermic animals that retain or dissipate heat depending on environmental and physiological conditions. They use metabolism for thermogenesis or vasomotor changes to perform heat exchange (148). Assessment of body temperature in pigs helps determine their health status non-invasively (127, 138). The ocular, nasal, and auricular zones are considered the main thermal windows, but they are not free of difficulties. In the case of the ocular window, the size of the orbital surface can affect temperature readings, and periocular structures like the eyelashes can occlude images. Likewise, animals tend to close their eyes when in agony, precluding the use of IRT. In the auricular zone, meanwhile, the conformation and position of the ear may restrict IRT's usefulness. Differences between breeds with erect or droopy ears can also cause temperature variations due toblood vessels' projection and hair's presence (149, 150).

The alternative proposed is to evaluate the temperature in broader regions like the back, flanks, thorax, or abdomen. A study of 99 Landrace x Yorkshire pigs by Feng et al. (119) found that shoulder, center-back, and rump temperatures (taken at a distance of 0.5 and 1 m) had an overall moderate to strong and positive correlation of 0.5-0.7 with rectal temperatures. Therefore, IRT can potentially evaluate the core temperature of animals. Similarly, a report on 91 newborn piglets captured back and flank temperatures at 11- time points between birth and 48 h postpartum. Due to the strong correlation (*r* = 0.82) with rectal temperatures found, the authors determined maximum surface temperature and rectal values, discovering that they were affected by time. At 25 and 30 min, respectively, those values decreased by 0.25 and 0.42°C. Moreover, they found that a maximum surface temperature of 30°C was indicative of rectal values < 32°C (65). What these authors observed would confirm that other regions may be related to thermoregulation processes. For example, local temperature in the nostril is affected by other factors, while regions such as the shoulder or back are known as a pathway to losing heat, then body temperature influences its values (104).

The thermal response of the back can also be used to indicate the mental state of animals. In 46 pigs involved in agonistic interaction, back and ear temperatures decreased significantly, by 0.9°C, during a conspecific confrontation event, and this variation had a moderate negative correlation with higher lactate (*r* = −0.49) and glucose (*r* = −0.32) levels, where temperature decreases were accompanied by increases in both endocrine markers (149). The branches of the vertebral arteries that supply the transverse spinal muscles are the biological foundation of these changes, as the branches of the spinal nerves are susceptible to sympathetic stimulation during stress responses, in which they also participate (112).

Other regions that have been suggested as thermal windows? are the gluteal and vulvar areas. Scolari et al. (151) mention that these areas (monitored from a distance of 0.61 m) tend to change the temperature during estrus. Specifically, the vulvar region increased its temperature by 1.5°C 12 h before ovulation due to the hemodynamic changes that occur during estrus. This was confirmed by Simöes et al. (152), who found that vulvar-gluteal temperatures (monitored from a distance of 1 m) increased during proestrus and 25 h before estrus but decreased by 1.1 ± 0.9°C 6 h after estrus. These changes suggest the possibility of adopting this window to predict estrus and increase reproductive success. In addition, a high success rate of IRT in detecting estrus and ovulation in 80 sows was achieved by assessing these conditions in conjunction with ultrasonic sensors (153).

Regardless of the region used as a thermal window, it is important to consider that environmental, technical, and individual factors can cause variations in the results (154). In the case of environmental factors, Basak et al. (155) determined the relation of temperature and humidity inside pig barns to surface temperatures measured on the right and left flanks, forehead, and back. They found an association between the temperatures of the barn and the body. In addition to these two variables, they concluded that other elements –wind speed, air pressure, body weight, and food consumption– require models to determine their influence on IRT. Those findings are similar to the observations by Barreto et al. (156), who found a positive correlation between solar radiation, environmental humidity, and wind with body surface temperatures.

Regarding technical factors, the distance between the camera and the animal, the angle of view, and the resolution of the thermographic image are the main ones (154). Leizi et al. (157), after evaluating the influence of camera angle on body surface temperature readings, determined differences >2°C between angles of 74° *vs*. 76°. A technical report on six pigs published by Banhazi et al. (158) stated that perpendicular images taken at angles ≤ 60° minimized the error percentage of readings and made it possible to detect a thermal response called a cooling effect 10–15 min after wetting, even though the percentage of the wetting effect was registered only during the first minute. Strategies for minimizing the effect of the angle of view include using IRT with kinetic sensors, as this reduced temperature differences in a febrile swine model from 2°C without the sensor to 0.03–1.2°C with it (157).

On the other hand, the distance between the camera and the animal also shows ample variation from one study to another that can affect thermographic readings. Playá-Montmany and Tattersall (159) found a negative and moderate correlation between ocular temperatures (*r* = −0.58) and distance. They observed less temperature variation at a distance of 1.5 m than at 10–15 m. This can be attributed to the dynamics of radiation which tends to disperse toward other objects in the environment, causing a reduction in the values registered by IRT, which is why some authors recommend that thermograms should not be taken at a distance >1 meter (129). However, others recommend distances of 60 (130) or just 20 cm and maintaining a position perpendicular to the body (155). Given this variation, studies must be conducted to identify how distance affects temperature readings, and establish a standard.

Finally, within the individual factors, the presence of hair has been shown to cause temperature variations as large as 0.2°C (23), while a dark-colored layer can increase heat retention by impeding dissipation (11, 24). Pigs, however, have only fine hair that, on the one hand, impedes heat retention (160) but, on the other, leaves them susceptible to sudden temperature decreases, above all in the neonatal stage.

## Future directions

Research into the use of IRT with swine seeks to confirm its applicability in new areas of study due to its information on physiological states and animal health. One possibility could be to implement IRT continuously to evaluate the temperature in real time on production units in both individual animals and entire herds and so determine the presence of lesions or infectious diseases (161, 162). Although these future applications would be the areas to be developed, it is necessary to establish the reliability of IRT at different body regions. For example, the ocular window has been shown to suffer less surface temperature variation than areas such as the back, where solar radiation, humidity, or dirt can alter the reading (107).

In this regard, IRT could aid in the early identification of sick animals that present clinical signs of fever (163). The presence of fever, and the vasomotor responses at the peripheral level t it generates, constitute an area of opportunity for applying IRT in farming systems since it can identify pathological processes based on changes in surface temperature (128). For example, Islam et al. (129) used IRT with piglets' head, body, and tail regions to detect signs of gastrointestinal infection caused by *Salmonella typhimurium* and *Escherichia coli*. They found increases in body surface temperatures at 24 h with a peak at 72 h (41° and 37.4°C, respectively; p < 0.005). In relation to the respiratory disease caused by *Actinobacillus pleuropneuomoniae* in pigs, thermography detected changes in ocular and auricular temperatures (an increase of up to 1.8° and 8.1°C, respectively, in sick pigs) 4-6 h before the appearance of the first clinical signs (142), while at the thoracic level, IRT achieved 100% specificity and sensitivity (Cl 95%: 69–100%) as a method for orienting lung biopsies (110). Determining such temperature increases could also be applied in pigs sent for slaughter to identify those with systemic infections and then program quarantine measures for them (164). However, it is important to emphasize that its use is recommended in conjunction with other innovative methods, such as accelerometers, to evaluate the activity of animals (165). Other diagnostic tools are computerized tomography (166), radio-frequency identification, and machine-learning technologies that can estimate animals' productive parameters (161).

Likewise, IRT monitoring may help reduce economic losses for producers while simultaneously procuring animal welfare by allowing early detection of diseased individuals (162) or recognizing injured animals during transport and the association that this might have on meat quality due to acute stress or dehydration (8, 162). Applying IRT prior to slaughter, for example, makes it possible to estimate levels of well-being (118). In that period, pigs are susceptible to increases in body temperature due to transport and environmental temperatures inside the vehicle (167). IRT could be applied during transport to identify states of fatigue or hyperemia caused by travel, as Warriss et al. (168) demonstrated in their study of 28 pigs in which increases in ear temperatures (from 27.3 to 35.0°C) had a strong-to-moderate correlation with higher blood temperatures (*r* = 0.71, p < 0.001) and creatine kinase (*r* = 0.55) and serum cortisol concentrations (*r* = 0.50). Because increases in body temperature and the activation of metabolic pathways can impact meat quality, IRT has been suggested as a way to determine animal welfare and predict pork quality (118), given that pre-slaughter stress involves a physiological response that alters thermoregulation and affects meat quality, as has been shown in cattle (169). In the case of pigs, an IRT study of the dorsal region from neck to rump of 500 pre-stunning animals successfully detected animals with defective carcasses. In pigs with skin surface temperatures above 32.2°C, 71% of 49 animals showed such defects as pale, soft, exudative meat (PSE, 6%) and dark, firm, dry meat (DFD, 22%) (130).

IRT may also make it possible to evaluate other key parameters, such as respiratory rates, based on changes in local thermal patterns in the nasal region. Indeed, this has been suggested to assess states of health continuously and non-invasively, and has been tested in cattle (24). The reproductive efficiency and fertility of breeding boars are affected by heat stress and its effects on spermatogenesis (170), a process that requires scrotal temperatures 4–6°C below the boar's body temperature to maintain semen quality and prevent infertility (171). Stravogianni et al. (172) evaluated this in five boars, reporting a strong, significant negative correlation (*r*^2^ > 0.5) between increases in scrotal temperature measured by IRT and a rapid, progressive reduction of sperm motility and velocity. These applications suggest that IRT could help detect thermal changes that alter fertility in boars. However, additional information is required on the sensitivity of this tool in such assessments.

The mammary window of sows is another region that could yield more sensitive information on states of health, as Rosengart et al. (173) argued in their study of 513 postpartum hybrid Viktoria sows. After birth, the piglets produced were divided into three groups: healthy, sick, and suspicious, by measuring the surface temperature of the sows' mammary glands. The authors found that the temperature of this window increased by 1.1°C in sick animals compared to healthy ones (*P* < 0.05). This could indicate a discrepancy in the sensitivity of other windows compared to the mammary region, which might mean that this anatomical region is ideal for determining pathological states due to its particular characteristics (108). This same window might also be useful for complementing diagnoses of conditions like postpartum dysgalactia, which causes a temperature increase in the mammary gland. Studies have proven that IRT can detect this pathology with a sensitivity of 94.4% and a specificity of 89.5%, so it may be able to corroborate that the anatomo-morphological characteristics of this window make it effective for evaluating states of health (174).

Finally, the vulvar region of sows has been studied due to the possibility that certain circulatory changes in those animals may indicate of their physiological condition; that is, IRT could help identify the estrus cycle and support assisted reproduction (175). Some authors suggest that a temperature increase of 2°C in this region can be indicative of estrus, which can be associated with the number of navicular cells (176). However, no reports have yet determined the sensitivity and specificity that would lead to validating this window in sows.

IRT has promising applications for the continuous monitoring of animal health. However, it is necessary to recognize that future research must consider some parameters that still need to be discovered„ such as the cut-off points for identifying when an animal is outside its thermo neutrality zone, a field that has only been studied in large ruminants (24). Likewise, improving a sensitive automatic technique that can reduce the influence of intrinsic and extrinsic factors that alter heat radiation must be considered to reduce the underestimation of thermal changes at critical points during the pig production process.

## Conclusions

Scientific evidence for the anatomical-physiological differences between piglets and adult hogs is clear. Because neonate piglets have immature thermoregulation systems and limited energy resources to compensate for ambient temperatures, their most important challenge is to prevent hypothermia during the drastic change from intrauterine to extrauterine conditions. In contrast, the adipose tissue deposits in adults can aid heat retention, though their scarce sweat glands impede dissipation. Hence, their greatest challenge is to avoid thermal stress. In light of this evidence, continuously evaluating these animals' thermal state is imperative.

Up to now, thermal windows like the armpit, back, shoulder, and snout are considered to have the greatest similarity to average rectal temperatures values, though the sites with reports of the best reliability and accessibility are the base of the ear, shoulder, and udder. The ocular and auricular thermal windows have emerged as viable options for evaluating thermal states in pigs and also for identifying stressful conditions since specific characteristics of this species make it possible to validate their effectiveness. The structure of the nasal window allows non-invasive evaluations of physiological parameters like respiratory rates, an obvious clinical application. On the other hand, the results obtained after evaluating the temperature of the dorsal, scrotal, mammary, and vulvar regions, suggest its efficacy in detecting defective carcasses and changes that alter fertility in boars, postpartum dysgalactia, and estrus, respectively. Scientific evidence for pigs, then, has demonstrated that the thermal windows analyzed herein aid in obtaining information on this species's general state of health.

Most studies have sought to validate thermal windows to ensure greater objectivity when using IRT with this species. However, to obtain accurate evaluations of the surface temperature, it must be considered that factors such as environmental temperature, the thickness of the hair or fur of each individual, and perfusion in the zones considered thermal windows cause considerable variations in the results.

## Author contributions

All authors contributed to the conceptualization, writing, reading, and approval of the final manuscript.

## Conflict of interest

The authors declare that the research was conducted in the absence of any commercial or financial relationships that could be construed as a potential conflict of interest.

## Publisher's note

All claims expressed in this article are solely those of the authors and do not necessarily represent those of their affiliated organizations, or those of the publisher, the editors and the reviewers. Any product that may be evaluated in this article, or claim that may be made by its manufacturer, is not guaranteed or endorsed by the publisher.
